# Effect of Vitamin D Supplementation on the Level of Circulating High-Sensitivity C-Reactive Protein: A Meta-Analysis of Randomized Controlled Trials

**DOI:** 10.3390/nu6062206

**Published:** 2014-06-10

**Authors:** Neng Chen, Zhongxiao Wan, Shu-Fen Han, Bing-Yan Li, Zeng-Li Zhang, Li-Qiang Qin

**Affiliations:** 1 Department of Nutrition and Food Hygiene, School of Public Health, Soochow University, 199 Renai Road, Suzhou 215123, China; E-Mails: 18862247673@163.com (N.C.); zhxwan@suda.edu.cn (Z.W.); sfhan@suda.edu.cn (S.-F.H.); 2 Medical College of Soochow University, 199 Renai Road, Suzhou 215123, China; E-Mail: bingyanli@suda.edu.cn; 3 Department of Labor Hygiene and Environmental Health, School of Public Health of Soochow University, 199 Renai Road, Suzhou 215123, China

**Keywords:** vitamin D, high-sensitivity C-reactive protein (hs-CRP), cardiovascular disease, meta-analysis

## Abstract

Vitamin D might elicit protective effects against cardiovascular disease by decreasing the level of circulating high-sensitivity C-reactive protein (hs-CRP), an inflammatory marker. Thus, we conducted a meta-analysis of randomized controlled trials to evaluate the association of vitamin D supplementation with circulating hs-CRP level. A systematic literature search was conducted in September 2013 (updated in February 2014) via PubMed, Web of Science, and Cochrane library to identify eligible studies. Either a fixed-effects or a random-effects model was used to calculate pooled effects. The results of the meta-analysis of 10 trials involving a total of 924 participants showed that vitamin D supplementation significantly decreased the circulating hs-CRP level by 1.08 mg/L (95% CI, −2.13, −0.03), with the evidence of heterogeneity. Subgroup analysis suggested a higher reduction of 2.21 mg/L (95% CI, −3.50, −0.92) among participants with baseline hs-CRP level ≥5 mg/L. Meta-regression analysis further revealed that baseline hs-CRP level, supplemental dose of vitamin D and intervention duration together may be attributed to the heterogeneity across studies. In summary, vitamin D supplementation is beneficial for the reduction of circulating hs-CRP. However, the result should be interpreted with caution because of the evidence of heterogeneity.

## 1. Introduction

Vitamin D is gaining increasing attention for its novel association with cardiovascular diseases (CVD). However, the effects of vitamin D supplementation on CVD prevention have not been completely determined because of the lack of randomized controlled trials (RCT) [1]. On the other hand, a number of studies have confirmed that C-reactive protein (CRP), an inflammatory marker, is a strong predictor of CVD [2,3,4]. Thus, it is likely feasible to prevent CVD with the help of CRP-reducing treatments. It is worthy of attention that vitamin D plays an important role in reducing inflammation [5,6], deducing us naturally to consider whether vitamin D supplementation protects against CVD incidence by decreasing the CRP level. Recently, a number of clinical trials assessing vitamin D supplementation on different populations have performed the determination of circulating CRP level. However, the sample size of these trials was small, the quality varied from low to high, and the results were inconsistent. Therefore, we conducted this meta-analysis of RCTs to assess the potential effects of vitamin D supplementation on circulating CRP level. High-sensitivity CRP (hs-CRP), which is essentially the CRP, is determined by means of a highly sensitive immunoturbidimetric assay. In this meta-analysis, hs-CRP was selected as the endpoint to minimize the likelihood of variation by determined methods.

## 2. Methods

### 2.1. Search Strategy and Study Selection

We followed the Preferred Reporting Items for Systematic Reviews and Meta-Analyses guidelines in the report of this meta-analysis [7]. We searched PubMed, Web of Science and Cochrane library through September 2013 (updated in February 2014) for relevant studies using the following search items: “vitamin D” or “cholecalciferol” in combination with “inflammation”, “high-sensitivity C-reactive protein”, “high-sensitive C-reactive protein” or “hs-CRP” with no restrictions. Reference lists and related records were manually reviewed. Only published studies were considered. We did not attempt to contact the authors for more information. Trials were included in the analysis if they were RCT and clearly reported the dosage of vitamin D supplementation, intervention duration and circulating hs-CRP level before and after the trials. Studies in which vitamin D was combined with calcium were included if the control group was also treated with the same dose of calcium. If more than one time point for the follow up was reported, the data from the longest period were used. Likewise, the data from the highest dose were used when more than one dose was administered for supplementation. In the case of multiple publications with duplicate/overlapped data for the same trial, the article with more detailed information was selected.

### 2.2. Data Calculation and Quality Assessment

Data extraction was performed independently by two investigators (N-C and LQ-Q). Discrepancies were resolved by discussion. The following data were extracted from eligible articles: the first author’s name, publication year, country of origin, design details, participant characteristics including sex, mean age, baseline level of plasma 25-hydroxyvitamin D_3_ [25(OH)D, a major circulating form of vitamin D] and health status, number of participants, intervention duration, daily dose of supplemental vitamin D, and circulating hs-CRP level. The value of hs-CRP level was converted into the same unit (mg/L). We assessed the methodological quality of each included trial by using the Jadad scale, which assigns scores for reported randomization, blinding, and withdrawals [8].

### 2.3. Data Synthesis and Statistical Analysis

The changes in hs-CRP levels in both intervention and control groups were calculated as the mean difference between the end and the baseline. If standard deviations (SDs) of these changes were not provided directly, then standard errors, median and quartiles were converted to SDs [9]. The mean baseline hs-CRP level for each study, if not provided, was obtained by combining the baseline values of the intervention and control groups, weighted by each group’s participant numbers.

The homogeneity across studies was tested using Cochran’s *Q*-test at *p* < 0.01, and quantified by the *I*^2^ statistic, which represents the percentage of heterogeneity that can be attributed to the variation across studies [10]. In the presence of significant heterogeneity, a random-effects model was used to calculate the pooled effect size; otherwise, a fixed-effects model was applied [11]. We further conducted pre-specified subgroup and meta-regression analyses to explore the possible sources of heterogeneity. We also performed a sensitivity analysis, in which a single trial was omitted each time and the effect size was recalculated to investigate its influence on the overall effect size.

Begg funnel plots and Egger regression test at the *p* < 0.10 were applied to assess the publication bias of this meta-analysis [12]. All of the data were analyzed by STATA version 11.0. *p* < 0.05 was considered significant unless otherwise specified.

## 3. Results

### 3.1. Search Results and Study Selection

**Figure 1:**
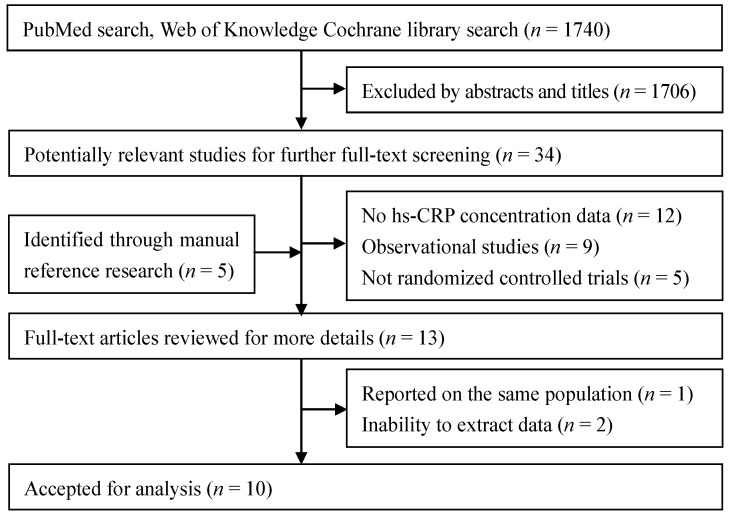
Flow chart of literature search and study selection.

A total of 1740 articles were found in our initial search and 1706 were excluded by screening abstracts or titles. After full-text review of the remaining articles and another 5 articles through manual reference research, we excluded 12 articles because hs-CRP data were unavailable. In addition, 9 observational studies and 5 trials without a specific RCT design were excluded. We excluded 3 more studies because one of them used the same population with an included trial and the other two did not provide the original statistics. A total of 10 RCTs were selected for the final analysis. A flow diagram showing the selection process is presented in Figure 1.

### 3.2. Study Characteristics

The characteristics of the included trials are presented in Table 1. Ten trials [13,14,15,16,17,18,19,20,21,22] were published from 2009 to 2014, in which four were conducted in Asia, 3 were conducted in Europe, 1 was conducted in Australia, and 2 were conducted in America. The sample size varied from 32 to 209, with a total of 924 participants. The treatment was double blind in eight trials and single blind in the two other trials. All of the trials had a parallel design and participants received treatment in capsules, pills, tablets, oil drops or a specially fortified yogurt drink. The duration of intervention lasted 8 to 48 weeks with a median of 24 weeks. The daily dose of supplemental vitamin D ranged from 400 to 7143 IU with a median of 4000 IU.

The diversity of participant characteristics was considerable in these trials. Seven studies enrolled men and women, whereas 3 others included only women [14,16,21]. Average age, if provided by studies, ranged from 25.1 to 84.5 years and the mean BMI ranged from 27.5 to 35.5 kg/m^2^. Except one trail with healthy and two with overweight/obese adults, other trials were conducted in pregnant women or participants with type 2 diabetes mellitus, coronary artery disease, polycystic ovary syndrome, insulin resistant condition or bedridden elder patients. Baseline circulating hs-CRP levels varied considerably from 1.71 to 22 mg/L with a median of 5 mg/L.

### 3.3. Main Analysis

**Figure 2:**
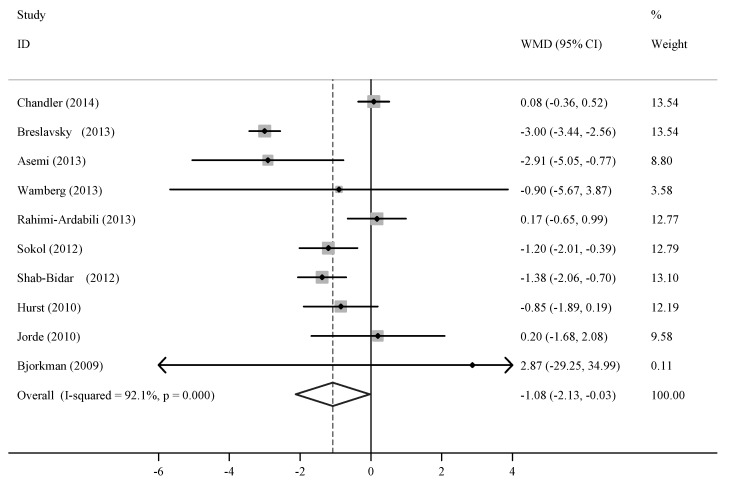
Meta-analysis of trials on vitamin D supplementation and circulating high-sensitivity C-reactive protein (hs-CRP) level.

Six out of the 10 trials reported a reduced level of circulating hs-CRP after vitamin D supplementation was administered. Since there was evidence of heterogeneity (*p* < 0.01, *I*^2^ = 92.1%), the random-effect model was applied. The pooled effect of vitamin D supplementation on circulating hs-CRP level was −1.08 mg/L (95% CI, −2.13 to −0.03; *p* < 0.01) compared with the control (Figure 2). This result indicated that supplemental vitamin D significantly decreased the circulating hs-CRP level.

**Table 1:** Characteristics of randomized controlled trials included in this meta-analysis.

Author	Year	Country	Status	Age (years)	BMI (kg/m^2^)	Sample Size		Baseline Serum Level		Vitamin D (IU/day)	Duration (week)	Jadad Scores
						Intervention	Control	25(OH) D (nmol/L)	hs-CRP (mg/L)			
Chandler [13]	2014	USA	Healthy black adults	51.0 (30–80)	31.0	78	71	NR	2.48	4000	12	4
Asemi [14]	2013	Iran	Healthy pregnant women	25.1 (18–35)	25.2	24	24	40.5	5.75	400	9	5
Breslavsky [15]	2013	Israel	Type 2 diabetes patients	66.3	29.3	19	13	29.8	5.00	1000	48	4
Rahimi-Ardabili [16]	2013	Iran	Polycystic ovary syndrome women	26.9 (20–40)	28.7	24	26	18.5	1.71	2500	8	5
Wamberg [17]	2013	Denmark	Obese adults	40.4 (18–50)	35.5 (>30)	22	21	<50	7.29	7000	26	5
Shab-Bidar [18]	2012	Iran	Type 2 diabetes patients	30–60	NR	50	50	NR	1.80	1000	12	2
Sokol [19]	2012	USA	Coronary artery disease patients	56.0	30.3	45	45	<50	22.00	7143	12	5
Jorde [20]	2010	Norway	Overweight outpatients	47.0	34.3 (28–47)	104	105	56.0	2.50	5741	48	2
Von Hurst [21]	2010	New Zealand	Insulin-resistant women	41.7	27.5	42	39	<50	2.45	4000	24	5
Bjorkman [22]	2009	Finland	Bedridden elderly inpatients	84.5 (>65)	NR	63	59	23.0	10.86	1200	24	4

NR, not reported.

### 3.4. Subgroup and Sensitivity Analyses

The results of subgroup analysis are presented in Table 2. Evidence of heterogeneity was also observed within any subgroup. Of note, vitamin D supplementation resulted in a significantly higher reduction of 2.21 mg/L (95% CI, −3.50, −0.92) in hs-CRP level among participants with baseline hs-CRP level ≥5 mg/L. Although the subgroup with low vitamin D supplement showed a significant decrease of hs-CRP, there is no statistically difference compared with its counterpart. We did not perform subgroup analysis according to mean age, BMI and baseline 25(OH)D because of insufficient data in some trials. Sensitivity analysis examining the impact of a single trial on the poled effect by omitting one trial in each turn yielded a range from −1.26 (95% CI −2.29, −0.23) to −0.70 (−1.35, −0.06).

**Table 2:** Results of subgroup analyses according to trial or participant characteristics.

Group	No	Net Change (95% CI)	*P-*heterogeneity	*I^2^* (%)	*P*-intergroup
Total	10	−1.08 (−2.13, −0.03)	<0.001	92.1	-
Baseline hs-CRP	-	-	-	-	0.023
≥5 mg/L	5	−2.21 (−3.50, −0.92)	0.004	73.8	-
<5 mg/L	5	−0.40 (−1.12, 0.31)	0.004	73.7	-
Vitamin D dosage	-	-	-	-	0.195
≥4000 IU/day	5	−0.49 (−1.21, 0.23)	0.060	55.4	-
<4000 IU/day	5	−1.69 (−3.27, −0.12)	<0.001	92.1	-
Intervention duration	-	-	-	-	0.559
≥24 weeks	5	−1.31 (−3.08 to 0.46)	<0.001	82.7	-
<24 weeks	5	−0.80 (−1.65 to 0.05)	<0.001	82.9	-

### 3.5. Meta-Regression Analysis

We performed univariate meta-regression analyses by using baseline hs-CRP level, vitamin D dosage and intervention duration as covariates. None of these covariates significantly influenced the pooled effect. However, baseline hs-CRP level, vitamin D dosage and intervention duration were highly associated with the pooled effect when they were introduced simultaneously to multivariate model; this result explained 100% of heterogeneity across studies.

### 3.6. Publication Bias

No sign of publication bias was observed when funnel plots were examined (plot not shown). The results of Begg’s (*p* = 0.79) and Egger’s (*p* = 0.75) tests did not also indicate the evidence of publication bias.

## 4. Discussion

To the best of our knowledge, this study is the first to perform a quantitative systematic analysis to evaluate the effect of vitamin D supplementation on circulating hs-CRP level. The present meta-analysis of 10 RCTs published in the last 5 years (2009–2014) showed that vitamin D supplementation significantly decreased the level of circulating hs-CRP by 1.08 mg/L. This effect was pronounced in participants with a baseline hs-CRP level ≥5 mg/L. The presence of heterogeneity across studies was attributed to the factors of baseline hs-CRP level, supplemental dose of vitamin D and intervention duration together.

Our findings for reducing circulating hs-CRP by vitamin D supplementation are biological plausible. Nuclear factor kappa B pathway (NF-κB)-dependent transcriptional activation is a major regulator of immune, inflammatory and stress responses. It has been known that NF-κB activation participate in endogenous CRP induction and activated NF-κB may enhance the effects of activator of transcription-3 (STAT3) [23]. In both lipopolysaccharide-stimulated murine macrophage cells and passively sensitized human airway smooth muscle cells, 1,25-dihydroxyvitamin D_3_ [1,25(OH)2D], a biologically active form of vitamin D, can inhibit NF-κB activation by upregulating the inhibitor of NF-κB (IκB-α) and reducing IkB-α phosphorylation [24,25]. In an animal study with diabetic mice, 25(OH)D intraperitoneal injection attenuated diabetic periodontitis by reducing serum TNF-α levels; the expressions of STAT3 and their phosphorylation were also inhibited in the gingival epithelia of mice, suggesting that STAT3 signaling is downregulated by 25(OH)D [26]. Thus, vitamin D supplementation, leading to elevation of 1,25(OH)_2_D and 25(OH)D, could suppress CRP by NF-κB and STAT3 signaling.

Cross-sectional studies have also suggested the potential anti-inflammatory effect of vitamin D in certain populations. Ngo studied 253 adults with mean hs-CRP level of 3.6 ± 4.0 mg/mL and found that serum 25(OH)D showed a significantly inverse associated with hs-CRP [27]. This inverse association was conformed in 147 morbidly obese participants whose hs-CRP levels ranged from 1.88 to 4.01 mg/L [28]. Interestingly, a recent cross-sectional study further observed a significantly inverse relation between 25(OH)D and CRP only in the group whose serum 25(OH)D was at level <52.5 nmol/L [29]. In fact, among 10 trials including this meta-analysis, 7 trials had baseline 25(OH)D <50 nmol/L and two did not provided baseline 25(OH)D.

However, some factors including baseline hs-CRP level and supplemental dose of vitamin D should be considered to explain these results. The participants involved in most of the trials are patients with more or less inflammatory complications. Because of keeping their medical treatment during the studies, their hs-CRP levels were likely maintained within the normal range or at a reasonable level. Hence, the circulating hs-CRP level in some trials with patients was even lower than that in trials with healthy adults. This phenomenon might limit the effect of vitamin D supplementation for further hs-CRP reduction. In the subgroup analysis, we really found that the beneficial effect of supplemental vitamin D was observed in participants with the high baseline hs-CRP (≥5 mg/L). Together with the 25(OH)D analysis, vitamin D supplementation could be more beneficial when circulating 25(OH)D level is low and inflammatory markers are high.

Diet and supplements are not the only source of vitamin D; another effective source of body vitamin D is from skin exposure to sunlight, which is remarkably influenced by seasonal variation, distance from the equator, sunscreen use and even the air pollution [30]. According to the United States Department of Agriculture, the dietary reference intake of vitamin D is 600 IU/day for 9–70-year-old population [31]. However, several studies have suggested that this value is inadequate for patients with inflammatory complications [32,33]. In the present study, sunlight-induced vitamin D was unlikely to influence the results since RCT study had the similar sunlight exposure in both vitamin D supplement group and the control group.

There are additional other limitations existing in this meta-analysis. Considerable heterogeneity across studies that made our findings complicated to interpret was the primary one. This is not surprising given the variation in study designs and characteristics of participants. Thus, evaluation of heterogeneity is a crucial part of any meta-analysis. In the present meta-analysis, meta-regression analyses showed that the baseline circulating hs-CRP level, supplemental dose of vitamin D and intervention duration together were the potential factors influencing the pooled effect. However, we cannot exclude other possible unmeasured confounders in this meta-analysis. In addition, the median and quartiles in some trials were converted into mean and their SDs for meta-analysis. The calculation for skewed distribution virtually reduced the precision. Finally, almost all of the trials were not designed to measure hs-CRP level as a primary outcome.

In conclusion, our findings demonstrated the significantly favorable effects of vitamin D supplementation on the level of circulating hs-CRP level, especially among participants with a baseline hs-CRP level ≥5 mg/L. However, the results should be interpreted with caution because of the evidence of heterogeneity. Additional well-designed RCTs are needed to determine the effect of supplemental vitamin D on the level of circulating hs-CRP.
